# RUNX1, an androgen- and EZH2-regulated gene, has differential roles in AR-dependent and -independent prostate cancer

**DOI:** 10.18632/oncotarget.2949

**Published:** 2014-12-10

**Authors:** Ken-ichi Takayama, Takashi Suzuki, Shuichi Tsutsumi, Tetsuya Fujimura, Tomohiko Urano, Satoru Takahashi, Yukio Homma, Hiroyuki Aburatani, Satoshi Inoue

**Affiliations:** ^1^ Department of Anti-Aging Medicine, The University of Tokyo, Bunkyo-ku, Tokyo, Japan; ^2^ Department of Geriatric Medicine, The University of Tokyo, Bunkyo-ku, Tokyo, Japan; ^3^ Department of Pathology, Tohoku University Graduate School of Medicine, Sendai, Miyagi, Japan; ^4^ Genome Science Division, Research Center for Advanced Science and Technology (RCAST), The University of Tokyo, Meguro-ku, Tokyo, Japan; ^5^ Department of Urology, Graduate School of Medicine, The University of Tokyo, Bunkyo-ku, Tokyo, Japan; ^6^ Department of Urology, Nihon University School of Medicine, Itabashi-ku, Tokyo, Japan; ^7^ Division of Gene Regulation and Signal Transduction, Research Center for Genomic Medicine, Saitama Medical University, Hidaka, Saitama, Japan

**Keywords:** RUNX1, androgen receptor, EZH2, prostate cancer

## Abstract

Androgen receptor (AR) signaling is essential for the development of prostate cancer. Here, we report that runt-related transcription factor (RUNX1) could be a key molecule for the androgen-dependence of prostate cancer. We found *RUNX1* is a target of AR and regulated positively by androgen. Our RUNX1 ChIP-seq analysis indicated that RUNX1 is recruited to AR binding sites by interacting with AR. In androgen-dependent cancer, loss of RUNX1 impairs AR-dependent transcription and cell growth. The *RUNX1* promoter is bound by enhancer of zeste homolog 2 (EZH2) and is negatively regulated by histone H3 lysine 27 (K27) trimethylation. Repression of RUNX1 is important for the growth promotion ability of EZH2 in AR-independent cells. In clinical prostate cancer samples, the RUNX1 expression level is negatively associated with EZH2 and that RUNX1 loss correlated with poor prognosis. These results indicated the significance of RUNX1 for androgen-dependency and that loss of RUNX1 could be a key step for the progression of prostate cancer.

## INTRODUCTION

Prostate cancer is the most frequently diagnosed cancer in men [1]. Androgen and its cognate receptor, the androgen receptor (AR), are involved in prostate oncogenesis through the transcriptional regulation of target gene networks [2]. To determine the genomic distribution of AR binding sites, it is important to consider binding sites for AR collaborating transcription factors such as FOXA1 [3, 4], NKX3-1 [5], GATA2, OCT1 [3] and members of the ETS family of transcription factors [6, 7].

Androgen-deprivation therapy is a standard treatment for prostate cancer and efficiently inhibits the growth of androgen-dependent tumors. Unfortunately, the majority of these cancers become refractory to hormone deprivation therapy and emerges as castration-resistant. Such castration-resistant prostate cancer (CRPC) is a significant clinical challenge, and investigation into the biologic mechanisms that contribute to tumor regrowth is of critical importance [1]. Mechanisms of castration-resistance can be grouped into two categories. The first is enhancement of AR signaling caused by mutation, amplification of the *AR* gene or other mechanisms that allow for the activation of AR even with castration levels of serum testosterone [1, 8-10]. The other group uses a mechanism to bypass AR signaling pathways [11], which allows cancer cells to survive in the absence of androgen-dependent or -independent AR activation. For example, cells that lack AR expression or had repressed AR signaling have been reported in large numbers of metastatic tumors derived from prostate cancer patients [12].

The runt-related transcription factor (RUNX) family consists of transcription factors such as *RUNX1, RUNX2,* and *RUNX3*. Each is capable of forming heterodimers with the common core-binding factor (CBF) β subunit [13]. RUNX-CBFβ heterodimers bind to their consensus target sequence, TGT/CGGT, and either activate or repress the transcription of target genes. Interestingly, each RUNX protein has differential function. RUNX1 is essential for hematopoiesis [14], RUNX2 for osteogenesis [15] and RUNX3 for neurogenesis [16]. In addition, *RUNX* genes are associated with different types of malignancy, and they function as both oncogenes and tumor suppressors in a tissue-specific manner [13].

In the present study, we demonstrated the molecular mechanism and clinical significance of RUNX1 expression in prostate cancer. By genome-wide analysis of AR-binding sites (ARBSs), we identified *RUNX1* as an AR target gene that is induced by androgens. RUNX1 expression is necessary for AR signaling and androgen-dependent prostate cancer cell proliferation. Furthermore, we showed that RUNX1 expression is correlated with a good prognosis for prostate cancer patients. Enhancer of zeste homolog 2 (EZH2), which is a major contributor to androgen-independent signaling in prostate cancer [17], represses *RUNX1* transcription, and the expression of RUNX1 is negatively associated with EZH2 in clinical samples. We also observed that repression of RUNX1 by EZH2 enhanced androgen-independent prostate cancer cell growth. Taken together, our results showed that RUNX1 plays a critical role in androgen-dependent and –independent prostate cancer cell growth. Our findings also highlighted the significance of RUNX1 loss in the progression of prostate cancer.

## RESULTS

### RUNX1 is a direct target of AR and interacts with AR

**Figure 1:**
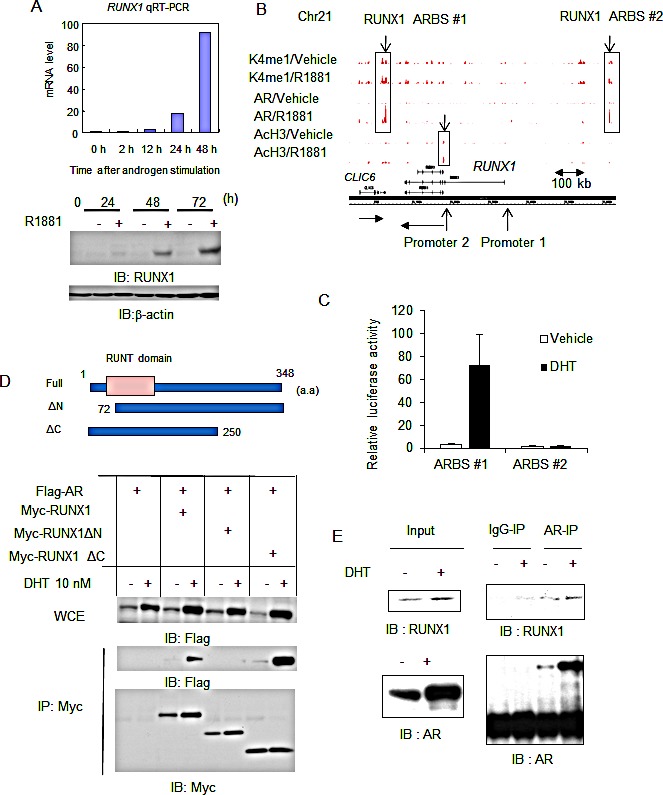
RUNX1, a direct target of AR in prostate cancer cells, interacts with AR androgen-dependently (A) RUNX1 is induced by androgen. LNCaP cells were treated with 10 nM R1881 or vehicle. Expression level of RUNX1 mRNA was measured by qRT-PCR. Data represent mean + s.d., n = 3. Western blot analysis of RUNX1 was performed. β-actin was used as a loading control. (B) ChIP-seq analysis of AR, AcH3 and K4me1 in the *RUNX1* locus. Two ARBSs were identified (ARBS #1 at 60 kb 3′-downstream region, ARBS #2: at 250 kb 5′ -upstream region). Two promoter loci and the direction of transcription were indicated by arrows. (C) Luciferase assay was performed in LNCaP cells. Luciferase vectors including ARBS #1 and #2 were used. Cells were treated with 10 nM DHT or vehicle for 24 h. Data represent mean + s.d., n = 3. (D) (Upper) Three Myc-tagged expression vectors of RUNX1 and deleted RUNX1 variants were constructed. (Lower) Androgen dependent interaction of RUNX1 with AR. 293T cells were transiently transfected with Flag-AR and Myc-RUNX1 and after 24 h incubation cells were treated with vehicle or 10 nM DHT for 24 h. Immunoprecipitation by Myc-antibody and western blot analysis of Flag and Myc was performed. (E) Endogenous interaction of AR with RUNX1. LNCaP cells were treated with vehicle or 10 nM DHT for 24 h. Cell lysates were immunoprecipitated by RUNX1 antibody. Western blot analysis of RUNX1 and AR was performed.

We previously reported the global analysis of androgen signaling by mapping ARBSs and androgen-regulated transcripts [18, 19, 20]. Using quantitative reverse transcriptase-PCR (qRT-PCR) analysis, we found that RUNX1 is highly induced by R1881 10 nM treatment in LNCaP cells (more than 50-fold following 48 h of treatment) (Fig.1A). RUNX1 protein levels were also increased as demonstrated by western blot analysis (Fig.1A). Two robust AR binding sites were identified around the *RUNX1* locus, one (ARBS #1) is 50 kb downstream and the other (ARBS #2) is 200 kb upstream (Fig.1B, Supplementary Fig.1A) by AR Chromatin immunoprecipitation and sequencing (ChIP-seq). In addition, RUNX1 upregulation by DHT treatment was also observed and this induction was inhibited by short interference RNA (siRNA) targeting AR-treatment (Supplementary Fig.1B, C). We also confirmed RUNX1 induction by androgen in other AR positive prostate cancer cells (Supplementary Fig.1D). Next, we investigated histone modification patterns in the *RUNX1* locus by ChIP-seq (Fig.1B, Supplementary Fig.1E). *RUNX1* has two distinct transcriptional start sites (TSSs: Promoter 1 and 2), and we found that promoter 2 is occupied with highly acetylated histone H3 by ChIP-seq analysis. Although both ARBSs are modified with monomethylation of histone H3K4, an active enhancer mark, we showed that ARBS #1 has a highly androgen-dependent transcriptional activity by luciferase assay (Fig.1C). Highly acetylated histone modification was observed only in the promoter 2 of RUNX1 (and not in that of CLIC6) around the ARBS#1, implicating that this region acts as an enhancer for RUNX1 induction (Fig.1B). These results suggest that *RUNX1* is directly regulated by AR binding.

Next, we analyzed the role of RUNX1 in prostate cancer cells and its association with AR. To examine whether AR interacts with RUNX1, we transfected 293T cells with expression vectors of Flag-tagged AR and Myc-tagged RUNX1 and its deletion mutants (Fig.1D, upper). We then performed immunoprecipitation by anti-Myc antibody and immunoblotted with anti-Flag antibody. We observed ligand dependent interaction of AR with RUNX1. When the N-terminal region of RUNX1 was deleted, this interaction was diminished, suggesting the N-terminal domain is responsible for AR interaction (Fig.1D, lower). We also showed the interaction of endogenous AR and RUNX1 in LNCaP cells (Fig.1E). Our results indicate that androgen-regulated RUNX1 interacts with AR that regulates gene transcription.

### Genome-wide analysis of RUNX1 binding sites reveals androgen-dependent recruitment of RUNX1 to ARBSs

**Figure 2:**
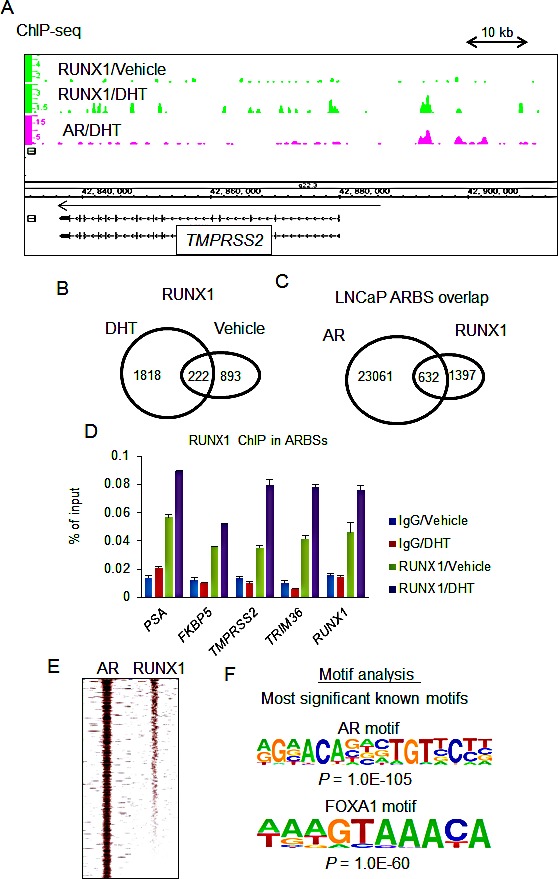
Genome-wide analysis of RUNX1 by ChIP-seq (A) Identification of RUNX1 binding sites by ChIP-seq. RUNX1 ChIP-seq analysis was performed in LNCaP cells. Cells were treated with vehicle or DHT for 24 h. Mapping of RUNX1 and AR binding sites in the vicinity of a representative androgen-regulated gene, *TMPRSSS2* on chromosome 21. (B) RUNX1 binding sites (*P* < 10^−5^) were determined by MACS. (C) Overlapping of ARBSs with RUNX1 binding sites. Venn diagrams depict the overlap of significant ARBSs with RUNX1 binding sites. (D) Recruitment of RUNX1 to androgen receptor binding sites (ARBSs). LNCaP cells were treated with vehicle or 10 nM DHT for 24 h. ChIP analysis was performed using a RUNX1-specific antibody. Enrichment of the ARBS was quantified using qPCR. Data represent mean + s.d., n = 3. (E) Heat map of RUNX1 binding tag intensity around peaks of AR binding sites (F) Motif analysis of RUNX1 binding sequences demonstrated the enrichment of AR and collaborative factor motifs. We analyzed 200-bp DNA sequences around RUNX1 binding peaks by using HOMER. Top two motifs by this analysis are shown.

We investigated the genome-wide binding of RUNX1 in androgen-dependent transcriptional regulation by ChIP-seq. LNCaP cells were treated with vehicle or DHT for 24 h and then ChIP was performed using a RUNX1 specific antibody. Enriched DNA was sequenced using a next generation sequencer and sequenced tags were mapped to the human genome (Fig.2A). We obtained 1125 significant RUNX1 binding sites in vehicle-treated cells and 2040 in DHT-treated cells using MACS (ref. 21, *P*-value < 1.0E-5, Fig.2B). We have previously reported significant DHT-dependent ARBSs (defined by MACS *P*-value <1.0E^−5^) in a previous publication [20]. As we expected, overlap of ARBSs with RUNX1 binding sites was observed and we validated androgen-dependent RUNX1 recruitment to representative ARBSs (Fig.2C-D, Supplementary Fig.2A). In addition, analysis of global RUNX1 ChIP-seq signals has revealed the distribution of RUNX1 bindings around the peak centers of significant ARBSs (Fig. 2E). The enriched motifs of transcription factors in RUNX1 binding sites were analyzed using HOMER [22]. Interestingly, the canonical RUNX1 binding motifs were not enriched (0.5% of all binding sites) although FOXA1 and AR motifs were enriched (Fig.2F), suggesting indirect binding of RUNX1 in ARBSs by binding to AR.

### Knockdown of RUNX1 inhibited AR binding and androgen-mediated gene induction

**Figure 3:**
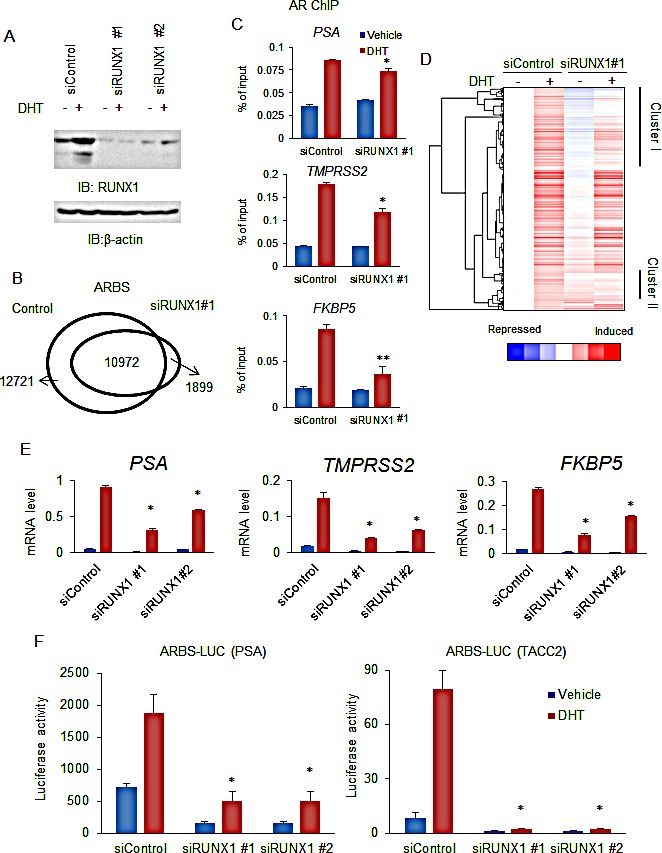
Knockdown of RUNX1 decreased the androgen-responsive transcriptional program (A) Knockdown of RUNX1 by siRNA transfection. LNCaP cells were transfected with siControl, siRUNX1 #1 and #2 (10 nM). Cells were transfected with vehicle or 10 nM DHT for 24 h. Western blot analysis of AR and RUNX1 was performed. β-actin was used as a loading control. (B) ChIP-seq analysis of AR binding with depleting RUNX1 expression. LNCaP cells were treated with siControl or siRUNX1 for 48 h. AR ChIP was performed after DHT treatment for 24h. ARBSs (Fold > 10, *P* < 10^−5^) were determined by MACS. (C) ChIP analysis of AR binding with depleting RUNX1 expression. LNCaP cells were treated with vehicle or 10 nM DHT for 24 h. ChIP analysis was performed using an AR-specific antibody. Enrichment of the ARBS was quantified using qPCR. Data represent mean + s.d., n = 3. (D) Global analysis of RUNX1 effects on androgen regulation of AR-binding genes. LNCaP cells were treated with siControl and siRUNX1 #1. After 48 h incubation, cells were treated with vehicle or 10 nM DHT for 24 h. Microarray analysis was performed and two clusters (Cluster I and II) were identified as genes positively regulated genes by RUNX1. (E) RUNX1 effects on androgen regulation of AR-binding genes. LNCaP cells were transfected with siControl or siRUNX1 #1 and #2 and then treated with 10 nM DHT or vehicle. Expression level of mRNA of genes in cluster I was measured by qRT-PCR. Data represent mean + s.d., n = 3. * *P* <0.01. (F) Effects of RUNX1 on transcriptional activity of AR. LNCaP cells were transfected with siControl or siRUNX1 #1 and #2. Luciferase vectors including PSA and TACC2-ARBSs were used. Cells were treated with 10 nM DHT or vehicle for 24 h. Data represent mean + s.d., n = 3. * *P* <0.01.

We next analyzed the effect of RUNX1 on AR binding by ChIP-seq. We treated LNCaP cells with short interference RNA (siRNA) targeting RUNX1 or control RNA (siControl) (Fig.3A). We performed AR ChIP after treating cells with DHT. In our ChIP and ChIP-seq study, the number of significant ARBSs obtained was decreased and enrichments of AR binding are inhibited by RUNX1 knockdown, suggesting a positive role of RUNX1 for AR binding (Fig.3B, C). The overlap of ARBSs with RUNX1 binding, and the reduction of ARBSs by RUNX1 knockdown were also observed in VCaP cells (Supplementary Fig.2B). These results indicate a significant role for RUNX1 recruitment to ARBS in AR binding.

Next, we examined the role of RUNX1 expression in androgen-mediated gene induction. We treated LNCaP cells with vehicle or DHT for 24 h after transfecting siRUNX1 or siControl. Then microarray analysis was performed to analyze the expression profile (Fig.3D). There were 895 genes selected as androgen-inducible (over 1.4-fold). Androgen-responsiveness of 620 genes (69%) was repressed by RUNX1 knockdown. Furthermore, qRT-PCR and luciferase analysis also showed that RUNX1 knockdown resulted in the inhibition of androgen-mediated gene and AR transcriptional activity in prostate cancer cells (Fig.3E, F, Supplementary Fig.3). These results indicate the positive effect of endogenous RUNX1 expression in androgen-mediated gene induction.

### RUNX1 promoter is repressed by trimethylation of H3K27 and negatively regulated by EZH2

**Figure 4:**
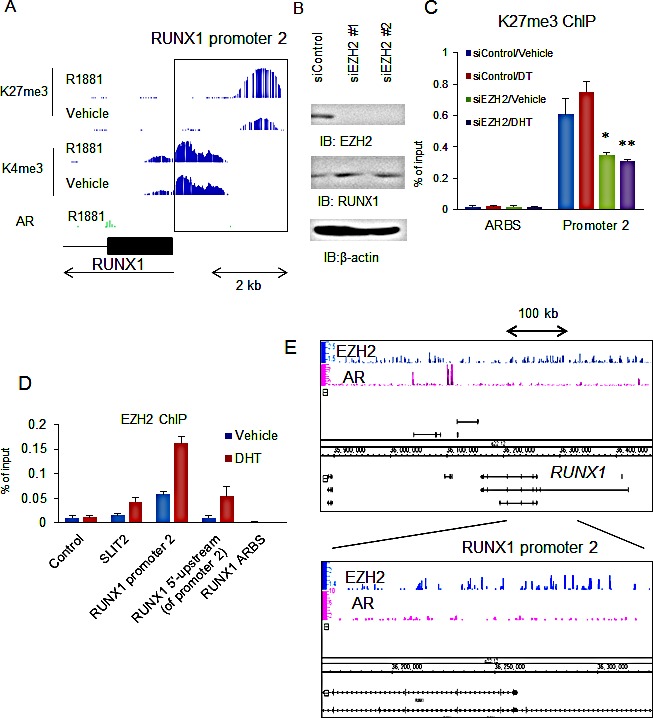
EZH2-dependent H3K27 methylation repressed RUNX1 expression (A) ChIP-seq analysis of the RUNX1 promoter region. LNCaP cells were treated with vehicle or 10 nM R1881 for 24 h. ChIP-seq analysis by K27me3 and AcH3 were performed. Signal distribution at the RUNX1 promoter is shown. (B) Effect of EZH2 knockdown. LNCaP cells were treated with siEZH2 #1 and #2 (10 nM). Western blot analysis of AR and RUNX1 was performed. β-actin was used as a loading control. (C) Analysis of K27me3 at the 5′-upstream region of RUNX1. LNCaP cells were treated with siEZH2 #1 or siControl. After 48 h incubation, cells were treated with vehicle or 10 nM DHT for 24 h. ChIP analysis was performed by using anti-K27me3 antibody. Enrichment of the 5′ upstream region of RUNX1 was quantified using qPCR. Data represent mean + s.d., n = 3. * *P* <0.05; ** *P* <0.01. (D) Analysis of EZH2 recruitment to 5′-upstream region of RUNX1. LNCaP cells were treated with vehicle or DHT. ChIP analysis was performed by using anti-EZH2 antibody. Enrichment of the promoter, 5′-upstream and ARBS regions of RUNX1 was quantified using qPCR. The SLIT2 promoter was used as a positive control for EZH2 recruitment. Data represent mean + s.d., n = 3. (E) ChIP-seq analysis of EZH2 binding at the RUNX1 promoter. LNCaP cells were treated with DHT for 24 h. Distribution of AR and EZH2 binding in RUNX1 regions is shown.

We analyzed whether RUNX1 is involved in androgen-independent prostate cancer development. By ChIP-seq analysis of histone modifications, we investigated the modification patterns of the *RUNX1* promoter (histoneH3 K4me3, K27me3, and AcH3). We found that the *RUNX1* promoter is occupied with modified histones not only the markers of activation, but also the repressive marker, H3K27me3 (Fig.4A). In prostate cancer cells, EZH2 overexpression is known to promote cancer progression [17]. EZH2 is a part of the polycomb complex that induces gene repression through histone H3K27 methylation. We observed that knockdown of EZH2 enhances the expression of RUNX1 (Fig.4B) and represses H3K27 methylation in the *RUNX1* promoter (Fig.4C). By ChIP assay we further investigated whether the *RUNX1* promoter is associated with EZH2 or not. SLIT2 is a representative target of EZH2 in prostate cancer and its promoter is occupied with EZH2 [23]. In LNCaP cells, we observed EZH2 recruitment, comparable with that of the SLIT2 promoter, to the 5′-upstream region and the promoter of RUNX1 (Fig.4D). By ChIP-seq, we further analyzed the regions occupied by EZH2 and identified multiple EZH2 binding sites in 5′-upstream region of the *RUNX1* gene (Fig.4E). Similar results were also observed in VCaP and DU145 cells (Supplementary Fig.4). These results suggest the regulation of RUNX1 by EZH2 dependent H3K27 methylation.

### Knockdown of RUNX1 repressed androgen-dependent, but enhanced androgen-independent prostate cancer cell proliferation

**Figure 5:**
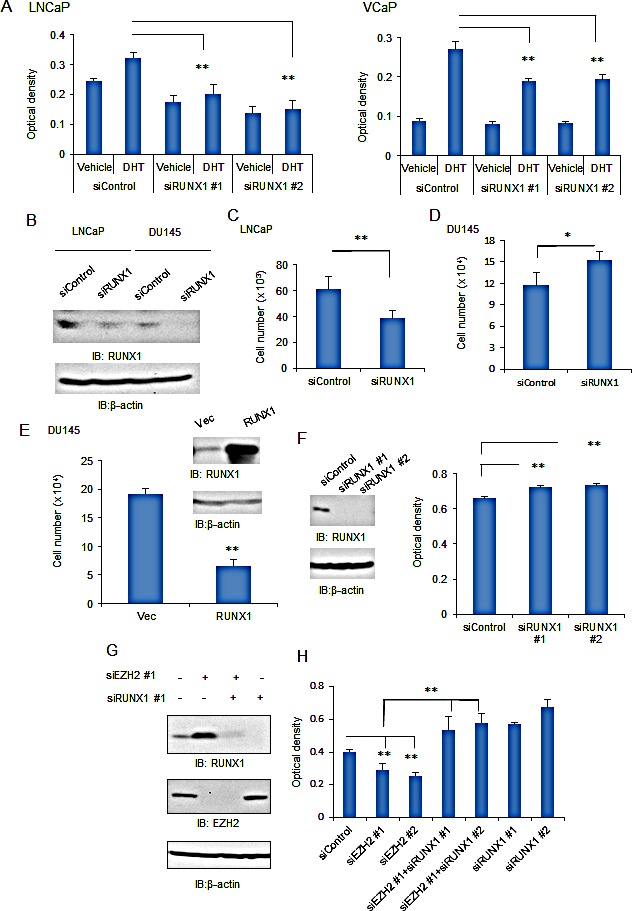
The role of RUNX1 in androgen-dependent and –independent prostate cancer cells (A) Knockdown of RUNX1 showed positive effects on androgen-dependent cell proliferation in prostate cancer. LNCaP or VCaP cells were transfected with siRUNX1 or siControl and then treated with vehicle or 10 nM DHT. Cell growth was evaluated at day 3 by MTS assay. Data represent mean + s.d., n = 4. ** *P* <0.01. (B) Knockdown of RUNX1 in both LNCaP and DU145 cells. Both cells were transfected with siControl or siRUNX1 #1 for 48 h. Western blot analysis of RUNX1 was performed. β-actin was used as a loading control. (C) LNCaP cells were transfected with siRUNX1 #1 or siControl and then the cell proliferation was assessed by cell counting on day 3. Data represent mean + s.d., n = 4. ** *P* <0.01. (D) DU145 cells were transfected with siRUNX1 #1 or siControl and then the cell proliferation was assessed by cell counting on day 3. Data represent mean + s.d., n = 4. * *P* <0.05. (E) Effect of RUNX1 overexpression in cell proliferation of DU145 cells. DU145 cells were transfected with RUNX1 expression vector or control. Western blot analysis of RUNX1 was performed. β-actin was used as a loading control. The cell proliferation was assessed by cell counting on day 3. Data represent mean + s.d., n = 4. ** *P* <0.01. (F) Knockdown of RUNX1 showed negative effects on androgen-independent cell proliferation in prostate cancer. DU145 cells were transfected with siRUNX1 or siControl. Western blot analysis of RUNX1 was performed. β-actin was used as a loading control. Cell growth was evaluated by MTS assay on day 3. Data represent mean + s.d., n = 4. ** *P* <0.01. (G) Knockdown of RUNX1 and EZH2. DU145 cells were transfected with siEZH2, siRUNX1 or siControl. Western blot analysis of RUNX1 and EZH2 was performed. β-actin was used as a loading control. (H) DU145 cells were transfected with siEZH2, siRUNX1 or siControl. Cell growth was evaluated by MTS assay on day 3. Data represent mean + s.d., n = 4. * *P* <0.05; ** *P* <0.01.

We explored the role of RUNX1 in prostate cancer cell proliferation. In LNCaP cells, we observed that androgen-dependent cell proliferation is inhibited by siRUNX1 transfection (Fig.5A). This effect of RUNX1 knockdown was also confirmed in VCaP cells. Next, we analyzed the role of RUNX1 in AR-negative prostate cancer cells. In DU145 cells, which represent androgen-independent cell proliferation, RUNX1 is expressed and effectively knocked down by siRUNX1 treatment (Fig.5B). By cell counting assays, we showed that proliferation of DU145 cells was promoted by RUNX1 knockdown in contrast to LNCaP cells, where proliferation is repressed (Fig.5C, D). The negative effect of RUNX1 on proliferation is also confirmed by overexpression of RUNX1 in DU145 cells (Fig.5E). We further analyzed whether this growth inhibitory effect of RUNX1 in androgen-independent cells was mediated by the function of EZH2 or not. We showed that RUNX1 knockdown by two different siRNAs promotes cell proliferation (Fig.5F). Surprisingly, the siEZH2-mediated growth inhibition was relieved by RUNX1 knockdown, suggesting EZH2 growth stimulatory effect is mediated partially at least by RUNX1 repression (Fig.5G, H). These results suggested that RUNX1 negatively regulates androgen-independent signaling for cell proliferation despite a positive effect on AR-mediated cell growth.

### RUNX1 is negatively associated with EZH2 expression in prostate cancer clinical samples

**Figure 6:**
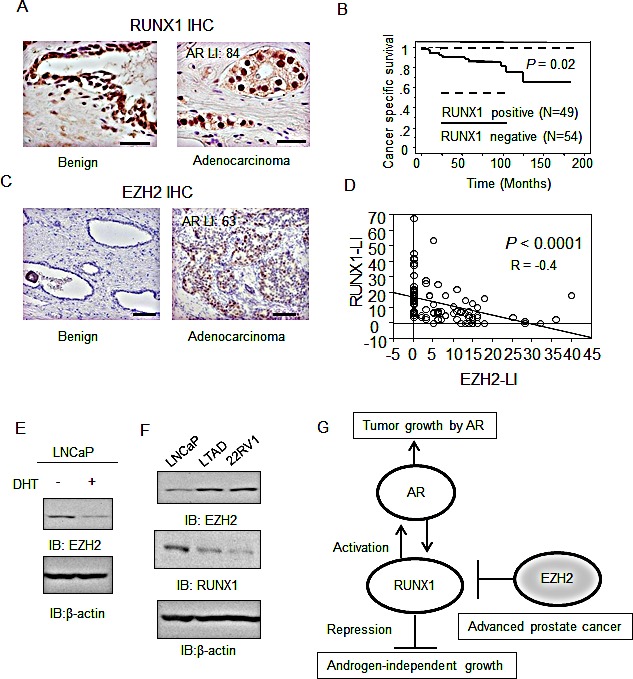
RUNX1 expression is negatively associated with EZH2 expression and loss of RUNX1 indicates a poor prognosis for prostate cancer patients (A) RUNX1 expression in prostate cancer. Immunohistochemistry (IHC) of RUNX1 in prostate cancer and benign prostate tissues (N = 103) was performed. Bar: 50 μm. (B) RUNX1 is a prognostic factor for prostate cancer patients. Kaplan-Meier analysis using the log-rank test was performed. (C) EZH2 is upregulated in prostate cancer. Immunohistochemistry of EZH2 in prostate cancer and benign prostate tissues (N = 103) was performed. Bar: 100 μm. (D) Negative correlation between RUNX1 and EZH2 expression levels. Regression analysis was performed to analyze the correlation. LI: labeling index. (E) Androgen-regulation of EZH2 in LNCaP cells. LNCaP cells were treated with vehicle or DHT for 24 h. Western blot analysis of EZH2 was performed. (F) Expression of EZH2 and RUNX1 in CRPC model cells. Protein expression of EZH2 and RUNX1 in LNCaP and CRPC model cells derived from LNCaP were analyzed by western blot analysis. (G) The role of RUNX1 in prostate cancer progression. RUNX1 promotes androgen-dependent prostate cancer cell proliferation by activating AR. However, in advanced prostate cancer, RUNX1 is downregulated epigenetically by EZH2. This repression would be correlated with another role of RUNX1 as a negative regulator of androgen-independent cell growth.

We performed immunohistochemical analysis to examine the protein levels of RUNX1 and EZH2 in clinical samples and to analyze the relationship between these factors (Fig.6A). In both prostate cancer and benign regions, RUNX1 is highly expressed in the nucleus, however, the labeling index (LI) of RUNX1 is decreased in prostate cancer tissues with a high Gleason score (Supplementary Table 1). Low RUNX1 expression in prostate cancer tissues was associated with poor cancer-specific survival of the patients (Fig.6B). In contrast, we observed strong expression of EZH2 in the nucleus of prostate cancer tissues, and weak expression in benign prostate tissues surrounding cancerous regions (Fig.6C). In addition, EZH2 expression is associated with poor prognosis of the patients (Supplementary Fig.5). Moreover, a strong negative correlation was observed between RUNX1 and EZH2 expression levels (R = −0.40, *P* < 0.0001, Fig.6D).

Additionally, we investigated the expression of RUNX1 and EZH2 in LTAD cells derived from LNCaP cells and 22Rv1 cells that were used as cell models for AR-positive CRPC. We observed EZH2 expression level was repressed by androgen treatment and long-term androgen depletion increased EZH2 expression (Fig. 6E, F). Conversely, RUNX1 expression is repressed by long androgen-depletion (Fig. 6F). Thus, we revealed a significant role of RUNX1 in androgen-dependent and androgen-independent cancer growth and demonstrated that RUNX1 loss could be a diagnostic tool for advanced prostate cancer as modeled in Fig.6G.

## DISCUSSION

In the present study, we first analyzed RUNX1 function in androgen-dependent prostate cancer growth because we found that RUNX1 expression is induced by androgen treatment. To analyze global transcriptional function, we then extensively mapped RUNX1 binding sites in the prostate cancer genome and identified RUNX1 is recruited to AR binding sites by direct interaction with AR. Our microarray analysis demonstrated that androgen-mediated gene induction is repressed by RUNX1 knockdown. Cell proliferation assays indicate the positive role of RUNX1 in androgen-dependent prostate cancer growth. Thus, the androgen-regulated RUNX1 positively enhances the transcriptional activity of AR.

Investigation of the role of *RUNX* genes in cancer started with the discovery of *RUNX1* as an important translocation breakpoint in leukemia [24], and dysregulation of RUNX-mediated gene expression has also been linked to the development and progression of malignancy [13]. Interestingly, RUNX1 was found in both normal and cancerous prostate cells [25]. The expression level and functions of RUNX1 in aggressive prostate cancer have not been fully investigated, although a single nucleotide polymorphism (SNP) in the *RUNX1* locus was found to be associated with the survival of prostate cancer patients, suggesting the significance of RUNX1 in prostate cancer [26].

Importantly, low expression of RUNX1 is associated with poor prognosis of the patients and high Gleason score in our clinical prostate cancer samples. This result raises the possibility that repression of RUNX1 could lead to promotion of tumor growth in advanced prostate cancer. Therefore, we analyzed the role of RUNX1 in androgen-independent cells. In AR-negative hormone-refractory prostate cancer, DU145 cells, RUNX1 knockdown enhances cell proliferation. These findings indicated that RUNX1 has dual roles in prostate cancer as a positive regulator by promoting AR binding and a negative regulator by modulating AR-independent signals. The poor outcome of prostate cancer with low expression of RUNX1 could be in line with this negative role of RUNX1 in prostate cancer growth. The downstream signals of RUNX1 in AR-negative prostate cancer cells require investigation to understand the specific role of RUNX1 in growth inhibition in AR-independent prostate cancer. Meanwhile, analyses of the RUNX1 transcriptional networks in previous studies have documented the activation of tumor suppressors, such as p19 [27] or p21 [28], or the inhibition of oncogenes by RUNX1 binding [29] for cancer progression. Therefore, AR-independent and RUNX1-regulated signals, which remain to be clarified, may be important for prostate cancer progression.

EZH2 plays a central role as a catalytic enzyme involved in histone H3 Lys 27 trimethylation (H3K27me3) which silences transcription [17]. The expression of EZH2 is correlated with CRPC progression [17, 30]. Tumor suppressors such as DAB2IP have been reported as EZH2 or PRC2 targets [31]. A recent report suggested an H3K27me3-independent mechanism of EZH2 by working as a co-activator of AR for gene activation in CRPC model cells [30]. Moreover, EZH2 is important in maintaining the pluripotency of embryonic stem cells or prostate cancer stem cells [32-34]. By investigating the histone modification of genes in prostate cancer, we found that the *RUNX1* promoter is occupied with H3K27me3 and that EZH2 is bound to the region. Clinical and cell-based experimental results indicate that RUNX1 and EZH2 are negatively correlated. Thus, we consider RUNX1 as a target of EZH2 in prostate cancer. Furthermore, the result of our cell proliferation assay indicates that RUNX1 repression is critical for the role of EZH2 for cell proliferation. Interestingly, we observed that EZH2 is androgen-repressed in LNCaP cells and upregulated in CRPC model cells such as LTAD and 22Rv1 cells. This result is in line with the past report in which EZH2 was found to be overexpressed in another CRPC cell line, abl cells [30]. We demonstrated that RUNX1 expression is downregulated in such cell lines. This RUNX1 repression may also be mediated by androgen-depletion because RUNX1 is a direct target of AR as shown in the present study. We expect that the transition of RUNX1 expression levels may be useful for a diagnostic marker for CRPC.

We unexpectedly identified a dual role for RUNX1 in prostate cancer progression. 1) To support AR activity and androgen-dependent cell growth, RUNX1 expression is required in AR-positive cells. 2) RUNX1 could exhibit EZH2-mediated tumor suppressive effects in prostate cancer development and progression, as suggested by clinical data. In AR-negative cell lines, RUNX1 knockdown enhances, while RUNX1 overexpression inhibits, the growth of prostate cancer cells. Our results indicated that RUNX1-mediated gene regulation, which is AR-independent, inhibits the cell growth. Based on these results, we speculate that RUNX1 may be essential for the survival of prostate cancer cells as AR is the key signal factor in clinical prostate cancer progression. In contrast, a reduction in RUNX1 expression, not overexpression, is required to prevent AR-independent growth inhibitory effect on prostate cancer cells. Further characterization of RUNX1-transcriptional networks and clinical analyses in context-specific conditions would reveal further details of prostate cancer progression.

In summary, this is the first report that both AR and EZH2 regulate RUNX1 and that RUNX1 was shown to have differential functions in androgen-dependent and androgen-independent prostate cancer cells. In addition, our clinical study indicated that low expression levels of RUNX1 would be an indicator of poor prognosis of prostate cancer patients. These findings suggest that the transition of RUNX1 expression could be a critical step for the progression of prostate cancer.

## METHODS

### Cell culture and reagents

Prostate cancer cell lines were purchased from ATCC (Manassas, VA). VCaP cells were grown in Dulbecco's Modified Eagle Medium supplemented with 10% FBS. LNCaP, DU145, and 22Rv1 cells were grown in RPMI medium supplemented with 10% FBS, 50 U/mL penicillin, and 50 μg/mL streptomycin. LTAD cells were grown in phenol-red–free RPMI medium supplemented with 10% charcoal-stripped FBS [19]. For androgen treatment, we treated cells with 1 or 10 nM R1881 or 10 nM dihydrotestosterone (DHT). The antibodies used in this study were RUNX1 (Upstate, Billerica MA), RUNX1 (Abcam, Cambridge, UK; ab23980), EZH2 (BD Biosciences, San Jose, CA; 612666), H3K27me3 (Abcam, ab6002). Other antibodies and reagents used have been previously described [18, 19].

### Small interfering RNA (siRNA)

For siRNA experiments, we purchased siRNAs targeting *EZH2 (#1: HSS176652, #2: HSS176653)* and a negative control siRNA from Life Technologies (Carlsbad, CA). The sequences of siRNA targeting RUNX1 are siRUNX1 #1: 5′-CCGCAGAACCAGAACGUUUUC-3′, and siRUNX1 #2: 5′-CCUGCGUUGGACCUUCCUUUU-3′ and we purchased them from Sigma Genosys (Spring, TX). Cells were transfected with RNA using an RNA interference (RNAi) reagent, Lipofectamine® RNAiMAX Reagent, (Life Technologies) 48–72 h before each experiment.

### Plasmid Construction

For construction of Myc-tagged RUNX1, the RUNX1 coding sequence (NM_001001890.2) was amplified by PCR and inserted into the *Eco*RI and *Xho*I sites of pcDNA3.0 including the Myc tag sequence at N-terminal end. For construction of the ARBS-luciferase vectors, the genomic regions (chr. 21: 35031129-35031554 (ARBS #1), and 35617483-35619831 (ARBS #2)) were amplified by PCR. Cloned ARBSs were inserted to the *Mlu*I and *Xho*I sites of the pGL3-promoter vector. PSA-LUC and TACC2-LUC have been described before [19].

### ChIP and ChIP-seq

ChIP and qPCR were performed as previously described [18, 19]. The fold enrichment relative to the IgG-IP control or input was quantified by quantitative PCR (qPCR) using SYBR Green PCR master mix and the ABI StepOne system (Life Technologies). The primer sequences for the detection of ARBSs by qPCR are listed below or described in previous studies [18, 19]. We performed AR, AcH3, K4me1, K4me3, RUNX1, H3K27me3 and EZH2 ChIP-seq in LNCaP cells using an Illumina Genome Analyzer or hiseq (Illumina, San Diego, CA) as described [20]. We also performed AR and RUNX1 ChIP-seq in VCaP cells. These sequence results have been deposited in NCBI's Gene Expression Omnibus (GSE62492). Sequence data Libraries were prepared according to Illumina's instructions. Unfiltered 36-bp sequence reads were aligned against the human reference genome (hg19 or hg18) using CASAVA v1.7 (Illumina). Signal scores of RUNX1 and EZH2 bindings were calculated using model-based analysis of ChIP-seq (MACS, ref. 21) and the threshold of binding sites was the *P*-value of < 1.0 E^−5^. Overlapping regions of RUNX1 and AR binding sites were determined by comparing the regions occupied by AR with those occupied by RUNX1. The regions shared by both peaks were designated as binding sites with overlapping AR and RUNX1. The other regions were classified as unique factor-specific binding sites, as shown in Fig. 2C.

Integrative genomic browser was used for visualization as described [19]. Motif analysis of binding sequences was performed using HOMER [22]. Primers are as follows: RUNX1 promoter 1 (forward: GCTGTGGGTTGGTGATGCT, reverse: GGACGAATCACACTGAATGCA), RUNX1 promoter 2 (forward: GCCAGCGAAGAGTTTCCTAGTC, reverse: GCGTGGCTGCTTTCAACTTT), RUNX1 5′-upstream (forward: GACGCTTGCTACAGACGTGA, reverse: CCACTGCAGGGGTAGTGATT), ARBS #1 (forward: TGATCAGATGCCCTGGAAATATAG, reverse: GCCGATGTCCAGTGTAAGCA), and ARBS #2 (forward: CTGGAACTTCTGTCCCCATC, reverse: TTTGCTGGTGATGGCAAATA).

### Cell proliferation assay

Cells were plated at 3 × 10^3^ cells per well in 96-well plates. For RNAi experiments, cells were transfected with siRNA 24 h after cell plating. The MTS [3-(4,5-dimethylthiazol-2-yl)-5-(3-carboxymethoxyphenyl)-2-(4-sulfophenyl)-2H-tetrazolium, inner salt] assay was performed using the CellTiter 96^®^ Aqueous MTS reagent (Promega, Madison WI), according to the protocol provided by the manufacturer. The experiment was performed five times. For cell counting, cells were trypsinized and counted using the trypan blue exclusion method to quantify cell viability.

### Western blot analysis and immunoprecipitation

For immunoprecipitation, 1 mg of cell lysate protein was incubated with anti-AR and anti-Myc antibody or normal rabbit IgG at 4°C overnight. The mixture of cell extract and antibody was then incubated with protein G-Sepharose beads (Amersham Biosciences, Piscataway, NJ) at 4°C for 2 h, and washed 4 times with NP-40 lysis buffer. The immunoprecipitated proteins were boiled for 5 min in Laemmli sample buffer and separated by SDS-PAGE. Immunoblotting was performed as described [19].

### Quantitative reverse transcriptase-PCR (qRT-PCR)

Total RNA was isolated using ISOGEN reagent. First strand cDNA was generated using the PrimeScript RT reagent kit (Takara, Kyoto, Japan). Expression levels were quantified by qPCR as previously described [19]. The primer sequences for RUNX1 are as follows: forward: ACTTCCTCTGCTCCGTGCT, reverse: GCGGTAGCATTTCTCAGCTC.

### Statistical analysis

For the cell proliferation assay, we analyzed 4 wells. For cell line experiments, statistical differences (*P*-values) among groups were obtained using a two-sided Student's *t*-test. All experiments were performed at least twice and similar results were obtained. *P*-values less than 0.05 were considered statistically significant. Statistical analyses were performed using GraphPad Prism 5 software (GraphPad Software, San Diego, CA) or MS Excel. The association between immunoreactivity and clinicopathological factors was evaluated using the Student's *t*-test, a cross-table with the chi square-test, or the correlation coefficient (*r*) and regression equation. The cancer-specific survival curves were generated according to the Kaplan-Meier method, and the statistical significance was calculated using the log-rank test.

### Microarray

For expression microarrays, the GeneChip Human Exon 1.0 ST Array (Affymetrix, Santa Clara, CA) was used according to the manufacturer's protocol. Data analysis was performed using the Affymetrix Microarray Suite software. To compare arrays, normalization was performed on data from all probe sets. For cluster analysis, we used Cluster 3 (downloaded from Eisen laboratory). The data have been deposited in NCBI's Gene Expression Omnibus and are accessible through GEO series accession number GSE62454.

### Luciferase assay

Cells were transfected with pGL3 vectors including ARBSs and tkTK-pRL by using the FuGENE HD reagent (Promega, Madison, WI). At 24 h after transfection, the cells were treated with 10 nM DHT or vehicle for 24 h, and the luciferase activities were determined as previously described [19].

### Prostate cancer cohort and immunohistochemistry (IHC)

We obtained prostate cancer samples (N = 103) from surgeries performed at the University of Tokyo Hospital (Tokyo, Japan). The Tokyo University ethics committee approved this study, and informed consent was obtained from each patient before surgery. The ages of the patients ranged from 52 to 78 years (mean, 67 years), and pretreatment serum prostate-specific antigen (PSA) levels ranged from 1.2 to 136 ng/mL (mean, 16.7 ng/mL). Other clinicopathological parameters are shown in Supplementary Table 1. Formalin-fixed tissues were embedded in paraffin and sectioned. A Histofine Kit (Nichirei, Tokyo, Japan), which employs the streptavidin-biotin amplification method, was used and the antigen-antibody complex was visualized with 3,3′-diaminobenzidine solution (1 mM 3,3′-diaminobenzidine, 50 mM Tris-HCl buffer [pH 7.6], and 0.006% H_2_O_2_). In an immunohistochemical analysis, the immunoreactivity was evaluated in more than 1000 carcinoma cells for each case. The percentage of immunoreactivity (LI: labeling index) was determined by trained pathologists [19]. Cases of LI > 10 were considered positive.

## SUPPLEMENTARY MATERIAL, FIGURES AND TABLES


