# Supplementary material for: RUNX1, an androgen- and EZH2-regulated gene, has differential roles in AR-dependent and -independent prostate cancer
Source: Oncotarget. 2014 Dec 10;6(4):2263–76. doi: 10.18632/oncotarget.2949 (PMC4385850; doi:10.18632/oncotarget.2949)
Supplement: Supplementary file 1 [file oncotarget-06-2263-s001.pdf]

RUNX1, an androgen- and EZH2-regulated gene, has differential roles in AR-dependent and -independent prostate cancer

Supplementary Material

Supplementary Table 1: Association between RUNX1 immunoreactivity and clinicopathological parameters in 103 human prostate carcinomas.

Value		n	RUNX1 LI (%)	*P* value
Age* (years)				0.59 (r = -0.054)
PSA* (ng / ml)				0.41 (r = -0.083)
Stage (Jewett Staging Sysyem)				
	B	33	13.9±2.6	
	C	59	11.8±1.4	
	D1	11	15.1±3.8	0.60
pT	pT1-2	34	16.2±2.7	
	pT3-4	69	11.2±1.2	0.55
pN	pN0	92	12.6±1.3	
	N1	11	15.1±3.8	0.71
Gleason score				
	2-6	40	16.1±2.4	
	7-8	30	13.2±1.6	
	8-10	33	7.4±1.6	**0.0059**

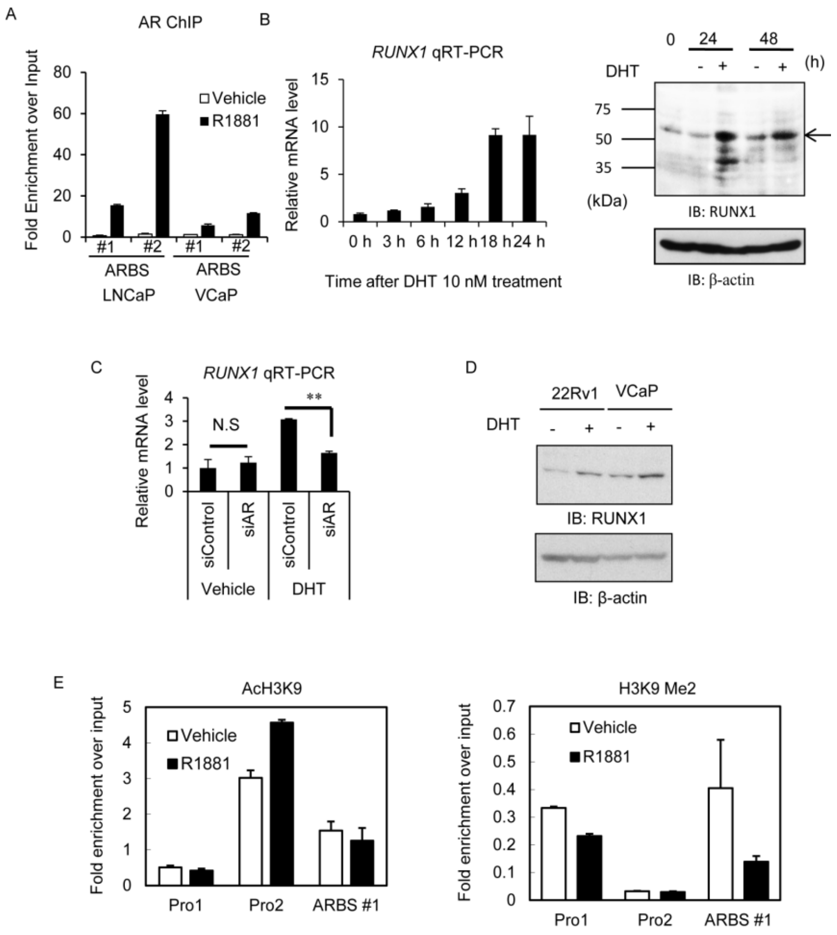

Supplementary Figure 1: AR recruitment and androgen regulation of RUNX1.

(A) ChIP analysis of AR at RUNX1 ARBSs identified by ChIP-seq. Both LNCaP and VCaP cells were treated with vehicle or 1 nM R1881 for 24 h. ChIP analysis was performed by using anti-AR antibody. Enrichment of RUNX1 ARBS #1 and #2 was quantified using qPCR. Data represent mean + s.d., n = 3.

(B) Induction of RUNX1 mRNA and protein by DHT treatment. LNCaP cells were treated with vehicle or DHT for 3, 6, 12, 18 and 24 h. Expression level of RUNX1 mRNA was measured by qRT-PCR (left). Data represent mean + s.d., n = 3. Western

blot analysis was performed to analyze RUNX1 protein (right).

(C) Androgen-dependent RUNX1 induction is inhibited by AR knockdown. LNCaP cells were transfected with siControl or siAR for 48 h. Cells were treated with Vehicle or 10 nM DHT for 24 h. Expression level of RUNX1 mRNA was measured by qRT-PCR. N. S: not significant, ** *P* <0.01.

(D) RUNX1 induction by androgen at other cell lines. AR positive prostate cancer cell lines (22Rv and VCaP cells) were treated with vehicle or 10 nM DHT for 24 h. Western blot analysis of RUNX1 was performed. Data represent mean + s.d., n = 3.

(E) Histone modification patterns at RUNX1 promoter. LNCaP cells were treated with vehicle or 1 nM R1881 for 24 h. ChIP analysis was performed using anti-AcH3K9 and K9me2 antibodies. Enrichment of hidtone modification at the RUNX1 promoter 1 (Pro1) and 2 (Pro2) and RUNX1 ARBS #1 was quantified using qPCR. Data represent mean + s.d., n = 3.

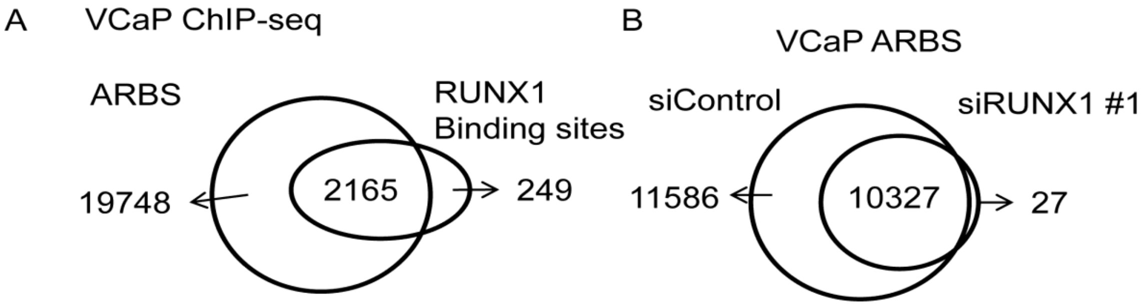

Supplementary Figure 2: ChIP-seq analysis in VCaP cells

(A) Identification of RUNX1 binding sites by ChIP-seq. RUNX1 ChIP-seq analysis was performed in VCaP cells. Cells were treated with 10 nM DHT for 24 h. RUNX1 binding sites ($P < 10^{-5}$) were determined by MACS. Overlapping of ARBSs with RUNX1 binding sites. Venn diagrams depict the overlap of significant ARBSs with RUNX1 binding sites.

(B) RUNX1 knockdown decreased AR binding. VCaP cells were treated with siControl or siRUNX1 #1 and then cells were treated with 10 nM DHT for 24 h. AR binding sites ($P < 10^{-5}$) were determined by MACS.

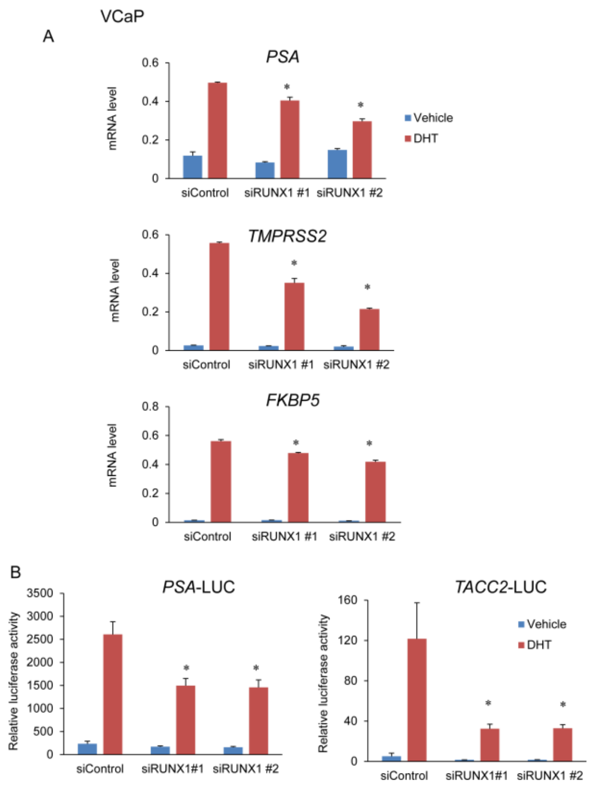

Supplementary Figure 3: Effects of RUNX1 in AR-mediated transcriptional activity in VCaP

(A) RUNX1 effects on androgen regulation AR regulated genes. VCaP cells were transfected with siControl or siRUNX1 #1 and #2 and then treated with 10 nM DHT or vehicle. Expression level of mRNA was measured by qRT-PCR. Data represent mean + s.d., n = 3. ∗ P <0.05.

(B) Effects of RUNX1 on transcriptional activity of AR. LNCaP cells were transfected with siControl or siRUNX1 #1 and #2. Luciferase vectors including PSA and TACC2-ARBSs were used. Cells were treated with 10 nM DHT or vehicle for 24 h. Data represent mean + s.d., n = 3. ∗ P <0.05.

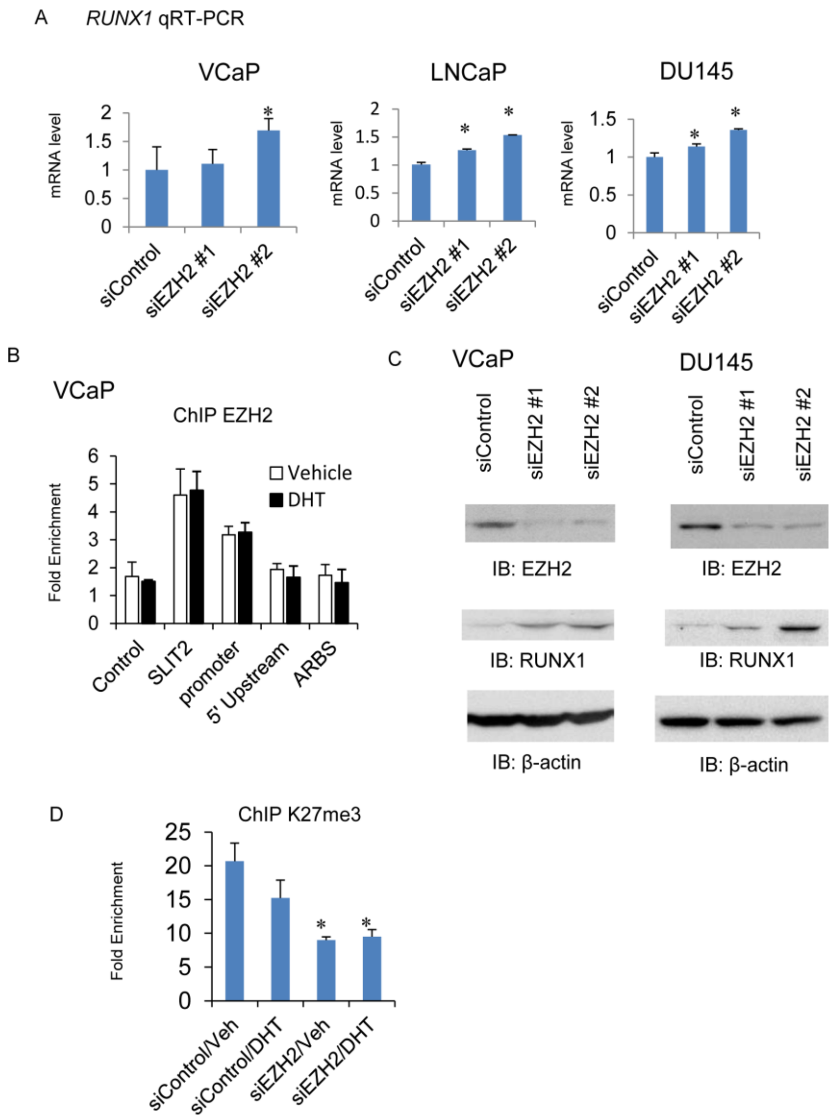

Supplementary Figure 4: Regulation of RUNX1 by EZH2 in prostate cancer cells.

(A) Effects of siEZH2 on RUNX1 mRNA expression in prostate cancer cells. LNCaP, DU145 and VCaP cells were transfected with siEZH2 for 48 h. Expression level of mRNA was measured by qRT-PCR. Data represent mean + s.d., n = 3. * P <0.05.

(B) Analysis of EZH2 recruitment to promoter region of RUNX1. VCaP cells were treated with vehicle or 10 nM DHT. ChIP analysis was performed using anti-EZH2

antibody. Enrichment of the promoter, 5’-upstream and ARBS regions of RUNX1 was quantified using qPCR. The SLIT2 promoter was used as a positive control for EZH2 recruitment. Data represent mean + s.d., n = 3.

(C) Effects of siEZH2 on RUNX1 protein expression in prostate cancer cells. VCaP and DU145 cells were transfected with siEZH2 for 48 h. Expression of RUNX1 was analyzed by western blotting.

(D) Analysis of K27me3 at the 5’-upstream region of RUNX1. VCaP cells were treated with siEZH2 #1 or siControl. ChIP analysis was performed by using anti-K27me3 antibody. Enrichment of histone modification at the 5’-upstream region of RUNX1 was quantified using qPCR. Data represent mean + s.d., n = 3. * P <0.05.

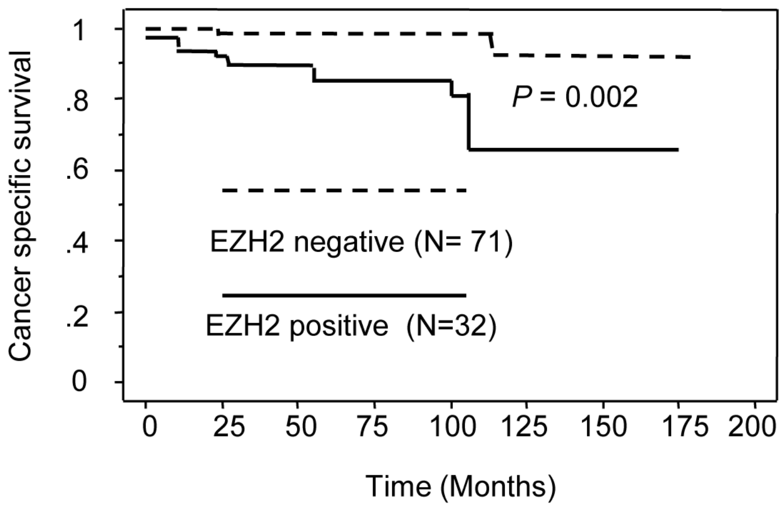

Supplementary Figure 5: Association of EZH2 expression with prognosis in prostate cancer patients. We performed EZH2 immunohistochemistry in Figure 6 (N = 103). Kaplan-Meier analysis using the log-rank test was performed.
